# Steady Quiet Asthma Without Biologics: One-Year Outcomes of Single-Inhaler Triple Therapy for Severe Asthma with Small Airway Dysfunction

**DOI:** 10.3390/jcm14155602

**Published:** 2025-08-07

**Authors:** Vitaliano Nicola Quaranta, Francesca Montagnolo, Andrea Portacci, Silvano Dragonieri, Maria Granito, Gennaro Rociola, Santina Ferrulli, Leonardo Maselli, Giovanna Elisiana Carpagnano

**Affiliations:** Respiratory Diseases, University of Bari, 70121 Bari, Italy; vitalianonicola.quaranta@policlinico.ba.it (V.N.Q.); francescamontagnolo17@gmail.com (F.M.); a.portacci01@gmail.com (A.P.); mariagranito1998@gmail.com (M.G.); gen.rociola98@gmail.com (G.R.); santina.ferrulli@gmail.com (S.F.); jaleo.maselli@gmail.com (L.M.); elisiana.carpagnano@uniba.it (G.E.C.)

**Keywords:** severe asthma, quiet asthma, inhaled triple therapy, extrafine

## Abstract

**Background:** Small airway dysfunction (SAD) plays a critical role in the management of severe asthma, particularly in patients at risk of requiring biological therapies (BTs). Short-term studies have shown that switching to single-inhaler triple therapy (SITT) with extrafine beclomethasone–formoterol–glycopyrronium improves outcomes and helps achieve quiet asthma, a state marked by symptom control, no exacerbations or oral steroids, reduced inflammation, and better small airway function. This study investigated whether, over one year, patients could maintain this state as Steady Quiet Asthma (SQA) and whether baseline measures could predict this sustained response. **Methods:** Twenty-six patients with severe asthma and SAD were transitioned from open triple-inhaler therapy to a closed, single-inhaler triple therapy containing extrafine beclomethasone–formoterol–glycopyrronium. Assessments at baseline (T0) and at one-year follow-up (T12) included clinical evaluations, spirometry, and impulse oscillometry, with a focus on Fres as a predictor for the need for BT. When prescribed, biologic therapies included mepolizumab, benralizumab, and dupilumab. **Results:** Of the 26 patients, 9 (34.6%) achieved SQA and did not require biologic therapy at the one-year follow-up, while 17 patients (65.4%) initiated biologic treatment. At T0, patients who required biologics had significantly higher median Fres (21 (19.47; 24.58) vs. 17.61 (15.82; 20.63); *p* = 0.049) compared to those who remained biologic-free. They also exhibited higher residual volume to total lung capacity ratio (%RV/TLC) values and lower forced expiratory volume in one second/forced vital capacity ratios (FEV_1_/FVC). At T12, patients spared from BT showed significant reductions in Fres (*p* = 0.014) and improvements in small airway function (difference in airway resistance between 5 Hz and 20 Hz (R5–20), forced expiratory flow between 25% and 75% of FVC (%FEF25–75), and better asthma control (ACT). In contrast, patients on BT demonstrated less favorable changes in these parameters. **Conclusions:** Baseline Fres, FEV1/FVC ratio, and %FEV25–75 are valuable predictors of achieving Steady Quiet Asthma (SQA) and sparing biologic therapy. These findings support the use of SITT in severe asthma and highlight the importance of early functional assessments to guide personalized management.

## 1. Introduction

Severe asthma, while affecting a small percentage of the overall asthma population, imposes a substantial clinical and economic burden [1]. Characterized by persistent symptoms, frequent exacerbations, and impaired quality of life, it remains challenging to manage despite advancements in therapeutic options [2]. Many patients fail to achieve adequate disease control, underscoring the critical need for treatment strategies that deliver sustained asthma management, reduce exacerbation risks, and alleviate disease-related limitations.

One therapeutic advance in severe asthma is the introduction of triple-inhaler therapy combining inhaled corticosteroids (ICSs), long-acting beta-agonists (LABAs), and long-acting muscarinic antagonists (LAMAs). This approach not only improves lung function and reduces exacerbations but also targets airway inflammation and remodeling by interfering with acetylcholine pathways. By blocking acetylcholine, produced by both parasympathetic nerves and non-neuronal cells, LAMAs help disrupt the cycle of inflammation and small airway dysfunction. Moreover, the integration of ICS/LABA/LAMA into a single inhaler simplifies treatment and enhances pharmacological synergy [3,4,5].

An important concept underlying the rationale for triple therapy is small airway dysfunction (SAD). SAD has emerged as a key contributor to disease severity, poor control, and persistent airflow limitations across asthma phenotypes [6,7]. As a recognized “treatable trait,” SAD represents a pivotal therapeutic target within precision medicine frameworks. This has generated interest in extra-fine formulations specifically designed to deposit medication in the peripheral airways and effectively address SAD.

Detecting and monitoring SAD in clinical practice requires sensitive tools. Impulse oscillometry (IOS) has demonstrated superior ability to detect SAD compared to traditional spirometry, enabling earlier recognition of peripheral airway impairment. By assessing resistance and reactance across different frequencies, IOS provides detailed insight into distal airway mechanics, thereby supporting targeted therapeutic adjustments [8,9].

Building on these concepts, this study investigates whether transitioning patients with severe asthma and SAD from open triple therapy to a single-inhaler extra-fine triple therapy can lead to a state of sustained clinical and functional control—termed Steady Quiet Asthma (SQA)—characterized by clinical stability, the absence of exacerbations or OCS use, and an improvement in small airway function. The study also aims to assess whether baseline functional parameters, particularly impulse oscillometry indices, can predict the achievement of SQA, thereby supporting a personalized, biologic-sparing treatment approach.

## 2. Materials and Methods

### 2.1. Study Design

This observational, prospective, and longitudinal study was conducted at the Severe Asthma Center of the Pulmonology Unit (UOC) at Policlinico of Bari, University of Bari Aldo Moro. The study enrolled 32 consecutive patients between 1 March 2023 and 1 October 2023, all of whom were followed at the severe asthma outpatient clinic. Of the 32 initial patients, only 26 completed the 12-month follow-up; the remaining 6 were lost to follow-up for non-clinical reasons (4 transferred to other specialist centers, 2 due to relocation). All non-completing subjects were contacted by telephone, and none reported significant clinical events.

We defined T0 as the baseline moment of the study, coinciding with the discontinuation of previous “open” triple therapy (ICS/LABA + LAMA in two separate inhalers), used for at least 12 months, and the initiation of a “closed” triple therapy in a single extra-fine inhaler containing beclomethasone dipropionate, formoterol fumarate, and glycopyrronium (BDP/FF/GLY). The new therapy was prescribed and initiated on the same day that baseline data were collected. Patients were re-evaluated after 12 months (T12).

### 2.2. Study Population

#### 2.2.1. Inclusion Criteria

- Age ≥ 18 years;
- Diagnosis of severe uncontrolled asthma according to Global Initiative for Asthma (GINA) step 5 criteria [10]
- Stable treatment for at least one year with triple inhaled therapy using two separate devices (ICS/LABA + LAMA);
- No prior exposure to biologic therapies;
- Presence of SAD phenotype, defined by at least one of the following:
  ○ Difference in resistance between 5 Hz and 20 Hz (R5–R20) > 0.07 kPa·L^−1^ s^−1^ on impulse oscillometry [11],
  ○ %FEF25–75 < 65% on spirometry [12].

#### 2.2.2. Exclusion Criteria

- Age < 18 years;
- Inability to perform pulmonary function tests;
- Documented non-adherence to therapy;
- Diagnosis of chronic obstructive pulmonary disease (COPD), cystic fibrosis (CF)-related bronchiectasis, allergic bronchopulmonary aspergillosis, mycobacterial infections, past tuberculosis, or pulmonary fibrosis;
- Current Aspergillus fumigatus infection;
- Prior exposure to biologic therapies was ruled out through a comprehensive review of each patient’s medical records and confirmation via clinical history at the time of enrollment.

**Figure 1:**
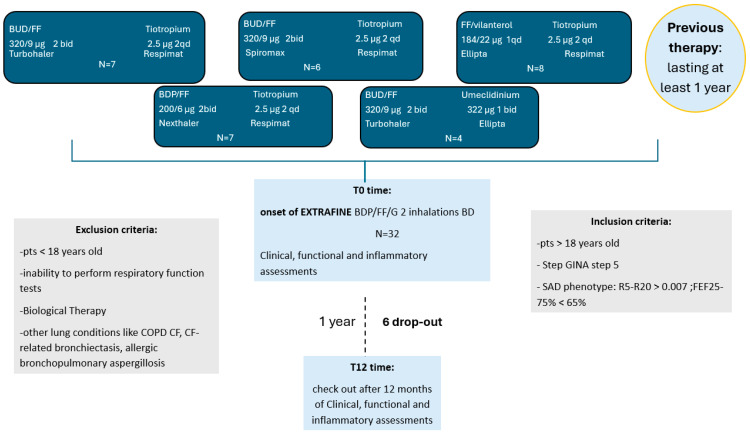
Flow chart of patient enrollment and follow-up. From the initial cohort of 32 patients assessed for eligibility, 26 completed the 12-month follow-up and were included in the final analysis. Six patients were excluded due to non-clinical reasons (relocation or referral to other centers). All patients transitioned from open triple therapy to single-inhaler extra-fine beclomethasone/formoterol/glycopyrronium and underwent assessments at baseline (T0) and at 12 months (T12). Abbreviations: BT, biologic therapy; SAD, small airway dysfunction; T0, baseline; T12, 12-month follow-up; BDP/FF/GLY, beclomethasone dipropionate/formoterol fumarate/glycopyrronium.

All patients signed written informed consent. The study was approved by the Ethics Committee of the A.O.U. Policlinico of Bari (protocol No. 6313, approved 4 March 2020) and conducted in accordance with the Declaration of Helsinki (Figure 1 illustrates the study enrollment and follow-up process).

### 2.3. Clinical Data and Patient Profile

At the T0 visit, in addition to respiratory parameters, the following were collected:

- Age, sex, body mass index (BMI), smoking status;
- Asthma duration, age at diagnosis, presence of atopy;
- Number of exacerbations and emergency room (ER) visits in the previous year;
- Use of OCS (number of cycles and annual dose), antibiotics, and Leukotriene Receptor Antagonist (LTRA);
- Comorbidities including eosinophilic granulomatosis with polyangiitis (EGPA), nasal polyposis, obstructive sleep apnea syndrome (OSAS), gastroesophageal reflux disease (GERD), vocal cord dysfunction, anxiety–depression syndrome questionnaires.

To assess asthma control and treatment adherence, all patients completed two validated tools:

- The Asthma Control Test (ACT) is a self-administered questionnaire of five multiple-choice questions assessing symptoms, rescue medication use, and activity limitations. The score ranges from 5 to 25; a score ≥ 20 indicates good clinical control. This tool is validated and considered a reliable surrogate for GINA-defined control [13].
- The Test of Adherence to Inhalers (TAI) includes 10 questions evaluating inhaler adherence and identifying three non-adherence profiles (erratic, deliberate, unconscious). It is specifically validated for asthma and COPD patients [14].

### 2.4. Lung Function

All patients underwent pulmonary function tests according to the ERS/ATS standards using the MasterLab Jaeger™ spirometer (Hoechberg, Germany). All lung function measurements—including spirometry, plethysmography, and impulse oscillometry—were performed during the same clinical session for each patient to ensure data consistency and reduce intra-individual variability. Tests included the following:

- Forced expiratory maneuvers to measure forced expiratory volume in one second (FEV_1_), forced vital capacity (FVC), forced expiratory volume in one second/forced vital capacity ratios (FEV_1_/FVC), and forced expiratory flow between 25% and 75% of FVC (FEF25–75%);
- Body plethysmography to determine total lung capacity (TLC), residual volume (RV), and residual volume to total lung capacity ratio (%RV/TLC), as well as total (Rtot) and effective resistance (Reff) [15].

### 2.5. Impulse Oscillometry (IOS)

Impulse oscillometry (IOS) was performed using the MasterScreen™ IOS (Jaeger, Hoechberg, Germany), following the protocol recommended by ERS for the forced oscillation technique [16]. The maneuver consisted of tidal breathing for approximately 30 s while the operator supported the patient’s cheeks to prevent distortion. IOS was performed during tidal breathing using at least three consecutive, technically acceptable maneuvers, as recommended by current ERS guidelines. The average value of these maneuvers was recorded and used for analysis. During the IOS procedure, the patient’s cheeks were manually supported by the operator to reduce upper airway artifacts and ensure an accurate measurement of respiratory impedance.

The parameters measured included the following:

- R5–R20: difference in resistance between 5 Hz and 20 Hz, an index of peripheral airway resistance. Values > 0.07 kPa·s·L^−1^ indicate SAD;
- X5: reactance at 5 Hz, reflecting elasticity of distal airways;
- Fres: resonance frequency, the point where impedance equals compliance;
- AX: area under the reactance curve, associated with peripheral lung compliance [17].

### 2.6. Exhaled Nitric Oxide (FeNO 50 and FeNO 350 mL/S)

Exhaled nitric oxide was measured using the FeNO+™ electrochemical analyzer (Medisoft-MGCD, Saint Paul, MN, USA) in line with ERS/ATS guidelines. Two flow rates were used:

- Fractional exhaled nitric oxide at 50 mL/s (FeNO at 50 mL/s), reflecting central airway inflammation;
- Fractional exhaled nitric oxide at 350 mL/s (FeNO at 350 mL/s), useful for estimating peripheral airway inflammation [18].

A positive intraoral resistance breathing technique was used in both tests to prevent nasal NO contamination and ensure data accuracy. Fractional exhaled nitric oxide (FeNO) was measured at two steady exhalation flow rates, 50 mL/s and 350 mL/s, each maintained for at least 6 s, in accordance with ATS/ERS guidelines. This dual-flow approach was used to assess both proximal (FeNO_50_) and distal (FeNO_350_) airway inflammation.

### 2.7. Statistical Analysis

All statistical analyses were conducted using IBM SPSS Statistics (version 24, IBM Corp., Armonk, NY, USA). Continuous variables were presented as the mean ± standard deviation (sd) for normally distributed data or as the median and interquartile range (IQR) for non-normally distributed data. The normality of data distribution was assessed using the Kolmogorov–Smirnov test. Categorical variables were reported as frequencies and percentages.

For within-group comparisons between the baseline (T0) and 12-month follow-up (T12), the paired Student’s *t*-test or Wilcoxon signed-rank test was used, depending on the distribution of the data. Comparisons between independent groups (e.g., patients requiring biologic therapy vs. those spared) were performed using the unpaired Student’s *t*-test or the Mann–Whitney U test for continuous variables and the chi-square or Fisher’s exact test for categorical variables.

Univariate logistic regression was conducted to identify potential predictors of achieving the “Steady Quiet Asthma” outcome. All variables with a *p*-value < 0.05 in univariate analyses were further evaluated for multicollinearity using variance inflation factor (VIF) analysis. Only non-collinear and clinically meaningful variables were entered into the final multiple logistic regression model to determine independent predictors of the outcome.

A receiver operating characteristic (ROC) curve was generated from the multivariate model to assess its discriminative performance. The area under the curve (AUC), along with the 95% confidence interval (CI), standard error (SE), and asymptotic significance (*p*-value), were reported. An AUC > 0.9 was interpreted as indicating excellent predictive power.

A *p*-value of less than 0.05 was considered statistically significant. No adjustments were made for multiple comparisons due to the exploratory nature of the study.

## 3. Results

### 3.1. Baseline Demographic and Clinical Characteristics

**Figure 2:**
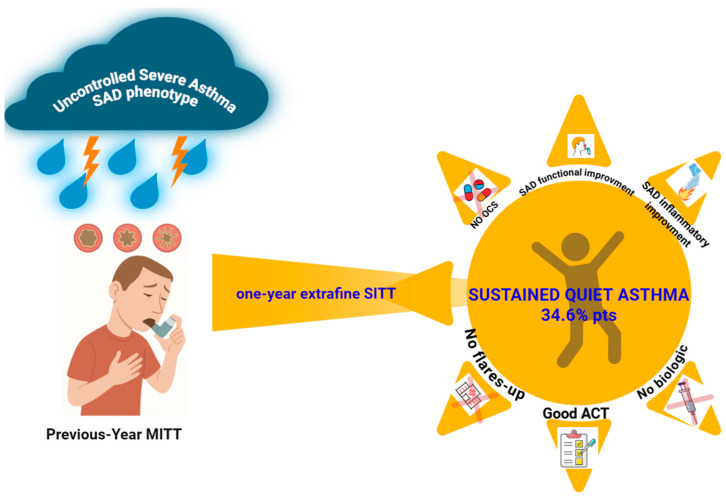
The study evaluates the 12-month effectiveness of transitioning patients with severe asthma and small airway dysfunction (SAD) from open triple-inhaler therapy to single-inhaler extrafine ICS/LABA/LAMA (BDP/FF/GLY). A focus is placed on the predictive role of baseline oscillometry—particularly the resonance frequency (Fres)—in sparing biological therapy. At follow-up, 34.6% of patients achieved sustained asthma control without requiring biologics, with significant improvements in small airway function and inflammation. Abbreviations: SAD, small airway dysfunction; ICSs, inhaled corticosteroids; LABAs, long-acting beta-agonists; LAMAs, long-acting muscarinic antagonists; BT, biologic therapy; IOS, impulse oscillometry system; Fres, resonance frequency; BDP/FF/GLY, beclomethasone dipropionate/formoterol fumarate/glycopyrronium; ACT, asthma control test.

An overview of the study design and major findings is provided in the graphical abstract (Figure 2). A total of 26 patients completed the 12-month follow-up. The mean age was 50.38 ± 14.56 years, with a slight female predominance (53.8%, n = 14). The average BMI was 26.26 ± 4.03 kg/m^2^. The cohort had a long-standing history of asthma, with a mean disease duration of 21.03 ± 13.33 years and a mean age at diagnosis of 28.65 ± 16.97 years.

During the year preceding enrollment, patients experienced an average of 3.00 ± 3.06 exacerbations, 0.26 ± 0.53 emergency room visits, and 1.65 ± 1.93 courses of OCS, with a cumulative annual prednisone-equivalent dose of 18.65 ± 12.92 mg.

Common comorbidities included gastroesophageal reflux disease (GERD; 26.9%), obstructive sleep apnea syndrome (OSAS; 23.1%), nasal polyposis (15.4%), and eosinophilic granulomatosis with polyangiitis (EGPA; 7.7%) (Table 1).

### 3.2. Clinical and Functional Outcomes Following 12 Months of Closed Triple Inhaler Therapy

At 12 months, significant clinical improvements were observed across multiple domains:

- OCS exposure: There was a significant reduction in the number of OCS cycles (median: 1 [IQR 0–2] to 1 [0–1]; *p* = 0.005) and in the cumulative OCS dosage (median: 25 [0–25] mg to 5 [0–12.5] mg; *p* = 0.001).
- Exacerbations: Annual exacerbation rates decreased significantly (median: 2 [1–4] to 2 [0–2]; *p* = 0.017).

From a functional perspective, the therapy yielded marked improvements in small airway parameters:

- %FEF25–75 increased from 48.50 ± 13.86% to 63.11 ± 12.13% (*p* < 0.001),
- Residual volume (RV) decreased from 177.94 ± 60.24% to 145.69 ± 38.82% of the predicted (*p* < 0.001),
- The RV/TLC ratio was reduced from 155.21 ± 30.34% to 131.11 ± 32.34% (*p* < 0.001),
- R5–R20 significantly decreased (from 0.14 [0.09–0.27] to 0.09 [0.07–0.14]; *p* = 0.031),
- Fres and X5 values also showed favorable changes, suggesting improved peripheral compliance.

In terms of airway inflammation, FeNO 350 values dropped significantly from 20.15 ± 13.17 ppb at baseline to 13.15 ± 7.17 ppb at follow-up (*p* = 0.010), indicating reduced distal airway inflammation (Table 2).

### 3.3. Subgroup Analysis: Patients Requiring Biologic Therapy Versus Those Achieving Sustained Quiet Asthma

**Figure 3:**
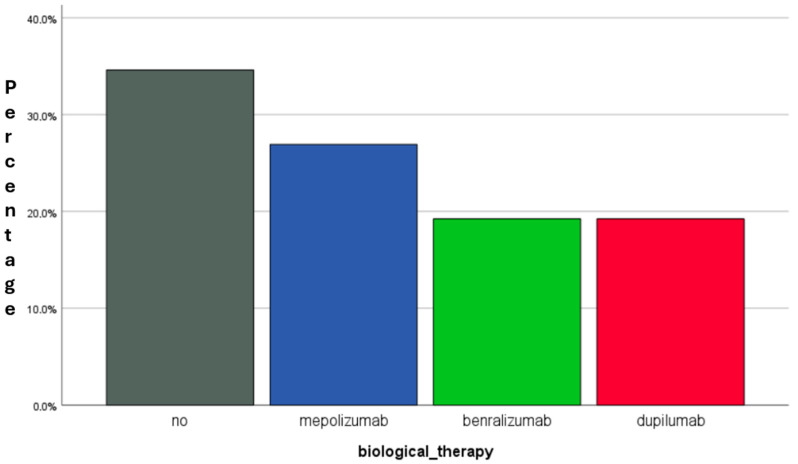
Distribution of patients by biological therapy requirements at the 12-month follow-up. Legend: of the 26 patients evaluated, 17 (65.4%) required escalation to biological therapy (BT), including mepolizumab (n = 7), benralizumab (n = 5), and dupilumab (n = 5). The remaining 9 patients (34.6%) maintained clinical stability without BT, defining the Sustained Quiet Asthma (SQA) group. This subgroup showed marked functional and inflammatory improvement with single-inhaler triple therapy” (SITT) alone. Abbreviations: BT, biologic therapy; SQA, Sustained Quiet Asthma; Fres, resonance frequency; FEV1/FVC, forced expiratory volume in 1 s/forced vital capacity ratio; RV/TLC, residual volume/total lung capacity ratio; %FEF25–75, forced expiratory flow between 25% and 75% of FVC.

By the end of the 12-month period, 9 patients (34.6%) achieved sustained clinical stability without requiring biologic therapy, constituting the “Sustained Quiet Asthma” (SQA) group. Conversely, 17 patients (65.4%) required the initiation of biologic therapy due to persistent clinical instability. The biologic agents prescribed included mepolizumab (n = 7, 26.9%), benralizumab (n = 5, 19.2%), and dupilumab (n = 5, 19.2%) (Figure 3).

At baseline, patients who ultimately achieved SQA demonstrated a more favorable small airway profile compared to those who required biologic therapy:

- Lower resonance frequency (Fres) values were observed (17.61 vs. 21.00 Hz; *p* = 0.049), indicating better peripheral airway function.
- The FEV1/FVC ratio was significantly higher (73.92% vs. 61.12%; *p* = 0.010), reflecting less airflow obstruction.
- Although not statistically significant, these patients also showed lower residual volume (RV) (3.18 vs. 3.65 L; *p* = 0.369) and lower RV/TLC ratio (150.35% vs. 157.79%; *p* = 0.593), suggesting reduced air trapping.

At follow-up, the following was observed:

- The SQA group demonstrated significant improvements in Fres (*p* = 0.014), R5–R20 (*p* = 0.031) and %FEF25–75 (*p* < 0.001), accompanied by enhanced asthma control and reduced OCS dependence.
- In contrast, the BT group showed more modest functional gains, with persistent peripheral airway dysfunction and a less pronounced recovery in the FEV1/FVC ratio (Table 3 and Table 4).

### 3.4. Predictors of Steady Quiet Asthma: Univariate and Multivariate Analysis

In the univariate logistic regression analysis, several factors were associated with the achievement of the *SQA* outcome. Notably, a lower number of OCS cycles in the previous year (OR: 0.124; 95% CI: 0.020–0.763; *p* = 0.024), lower cumulative OCS dose (OR: 0.923; 95% CI: 0.856–0.995; *p* = 0.036), and higher values of %FEV_1_ (OR: 1.153; 95% CI: 1.029–1.291; *p* = 0.014), %FVC (OR: 1.143; 95% CI: 1.013–1.290; *p* = 0.030), %FEV_1_/FVC (OR: 1.156; 95% CI: 1.012–1.320; *p* = 0.033), and %FEV25–75 (OR: 1.098; 95% CI: 1.001–1.201; *p* = 0.046) were significantly associated with the outcome. To address potential collinearity among these variables, we performed a multicollinearity analysis using the variance inflation factor (VIF). Variables with significant overlap were excluded. In the final multivariate logistic regression model, none of the selected predictors retained statistical significance, although OCS cycles and %FEV_1_/FVC showed trends toward associations (Table 5).

**Figure 4:**
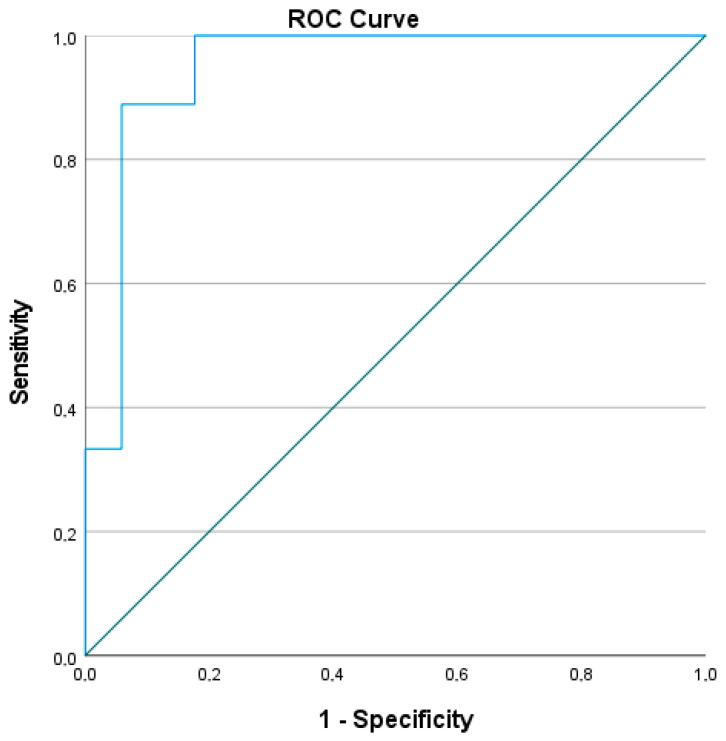
Predictive value of baseline parameters for achieving Steady Quiet Asthma. Abbreviations: AUC, area under the curve; SE, standard error; CI, confidence interval; Sig., significance level (*p*-value). An AUC of 0.948 (95% CI: 0.862–1.000; *p* < 0.001) indicates excellent discrimination of the model. Receiver operating characteristic (ROC) curve for the multivariate logistic regression model predicting Steady Quiet Asthma (SQA).

The ROC curve derived from the multivariate logistic regression model evaluating the predictive value of baseline parameters for achieving SQA showed excellent diagnostic accuracy. The area under the curve (AUC) was 0.948, with a standard error of 0.044, and the asymptotic significance was *p* = 0.000, indicating strong predictive capability. The 95% confidence interval ranged from 0.862 to 1.000, further confirming the robustness of the model (Figure 4).

## 4. Discussion

This study highlights the effectiveness of transitioning patients with severe asthma and small airway dysfunction (SAD) from open triple-inhaler therapy to a single-inhaler formulation of beclomethasone–formoterol–glycopyrronium. Building on our previous work, where we first introduced the concept of quiet asthma, a state of clinical and functional stability achieved after just 3 months of optimized inhaled therapy in patients with uncontrolled severe asthma and SAD [8], and we now demonstrate that this condition was sustained over one year in 34.6% of patients, who avoided escalation to biological therapy (BT).

A key finding is the identification of *SQA* as a multidimensional composite outcome achieved through inhaled therapy alone, defined not only by clinical stability but also by measurable improvements in small airway function (e.g., reductions in resonance frequency [Fres] and R5–R20, increases in %FEF25–75) and reduced peripheral inflammation, assessed via FeNO at 350 mL/s. Unlike the conventional concept of clinical remission [19], typically reserved for patients on biologics and lacking SAD or inflammatory domains, Steady Quiet Asthma offers a more comprehensive, inhaler-based target for the pre-biologic phase of asthma management. This novel concept prompts comparisons with established definitions of asthma remission, particularly those associated with biological therapy. Conventional remission is typically defined by the absence of exacerbations and oral corticosteroid use, improved symptoms, and sometimes normalized lung function—mostly in patients receiving biological therapy [2]. In contrast, SQA, as defined in our study, is a multidimensional target achievable with inhaled therapy alone. It includes clinical stability, objective improvements in small airway function, and reduced distal inflammation (FeNO). Unlike traditional definitions that rely mainly on symptom control and spirometry, SQA integrates oscillometry and inflammatory markers, offering a more comprehensive and practical goal in the pre-biological therapy phase. The single-inhaler triple therapy used in this study, which delivers extra-fine particles (<2 µm) to improve distal deposition [20], is supported by preclinical data showing synergistic bronchodilation via glucocorticoid receptor activation and β2-adrenoceptor Gsα stimulation, engaging the cyclic AMP–PKA pathway [21]. While these mechanisms are biologically plausible, they still require clinical validation. In our population, the switch from multiple-inhaler triple therapy (MITT) to single-inhaler triple therapy (SITT) was associated with durable improvements in asthma control, SAD parameters, and FeNO reductions. However, we were unable to distinguish whether these outcomes resulted primarily from improved adherence due to regimen simplification or from the pharmacodynamic synergy of the fixed combination; likely, both contributed. While simplified regimens have been shown to improve adherence and inhaler technique, the extrafine formulation used in SITT may also enhance peripheral drug deposition and deliver synergistic bronchodilation via glucocorticoid receptor activation and β2-adrenoceptor signaling. This pharmacologic synergy has been demonstrated in preclinical ex vivo models of human airway smooth muscle [20]. However, these findings should be interpreted cautiously, as clinical evidence directly confirming this mechanism in vivo remains limited. It should be noted that the absence of a control arm, such as continued open triple therapy, limits the ability to draw definitive causal inferences regarding the superiority of SITT. While the magnitude and consistency of improvements observed across clinical, functional, and inflammatory domains suggest a true therapeutic benefit, we cannot fully exclude the potential contribution of natural disease variability or regression to the mean.

In our logistic regression analysis, several baseline clinical and functional parameters were significantly associated with the achievement of Steady Quiet Asthma (SQA). Univariate analysis identified a lower oral corticosteroid (OCS) burden—both in terms of the number of cycles and cumulative dose—as well as higher values of %FEV_1_, %FVC, %FEV_1_/FVC, and %FEV25–75, highlighting the combined importance of small and large airway function in supporting sustained disease control. Although resonance frequency (Fres) did not reach statistical significance (*p* = 0.081), its inverse association with SQA aligned with the expected pathophysiological relevance of small airway dysfunction.

To refine these observations, we conducted a multivariate logistic regression analysis including all variables that were significant in the univariate phase. After adjusting for collinearity, no single parameter maintained statistical significance, underscoring the difficulty of isolating individual predictors in a condition as multifactorial as severe asthma. However, the composite predictive power of the model remained robust: the ROC curve analysis yielded an AUC of 0.948, demonstrating excellent overall discriminative ability. This suggests that while no isolated variable can independently explain the achievement of SQA, the interplay between clinical and functional factors forms a reliable predictive profile.

Importantly, patients who achieved Steady Quiet Asthma were also characterized by the absence of previous OCS use and more favorable baseline airway function—particularly higher %FEV25–75 and FEV_1_/FVC ratios, suggesting that baseline physiology contributed alongside the pharmacological effects of SITT.

Crucially, baseline functional parameters emerged as predictors of the treatment response: patients achieving *SQA* had significantly lower Fres and higher FEV1/FVC ratios at baseline, suggesting milder SAD and better preserved large airway function, whereas those progressing to BT exhibited higher Fres, lower FEV1/FVC, and trends toward increased air trapping, highlighting the importance of baseline stratification. The heterogeneity of the BT subgroup, in terms of both the type of biologic prescribed and the individual clinical indications, makes it difficult to clearly define shared characteristics or response patterns within this group. These results are consistent with a recent systematic review and meta-analysis in *The Lancet Respiratory Medicine* [22], which reported that clinical remission in patients treated with biologics was achieved in 38% of cases using a three-domain definition (absence of OCS, exacerbations, and symptoms), dropping to 30% when lung function normalization was included. Notably, the review included patients with type 2 inflammation and moderate-to-severe asthma, many of whom had long-standing disease and elevated symptom burden; remission was less likely in patients with lower baseline FEV1, longer disease durations, high symptom burdens, obesity, and depression, factors also seen in our non-responder group. Our remission rate is also in line with findings from the TRIMARAN and TRIGGER trials, which used the same extra-fine BDP/FF/GLY formulation and showed significant improvements in lung function and exacerbation reduction, specifically in patients with uncontrolled moderate-to-severe asthma who remained symptomatic despite medium or high-dose ICS/LABA therapy. These trials enrolled individuals with a history of frequent exacerbations and evidence of airflow limitation, closely resembling the clinical profile of our study population [3]. Furthermore, the recent SANI study confirmed that 34.5% of severe asthma patients treated exclusively with ICS/LABA/LAMA achieved full clinical remission, and another 34.5% achieved partial remission, in a cohort composed of GINA step 5 patients with persistent symptoms and frequent exacerbations despite high-dose ICS/LABA therapy. These findings further support the efficacy of optimized inhaled therapy without biologics in a population comparable to our own [23]. All patients in our study began with poorly controlled asthma and significant SAD, as evidenced by low ACT scores and impaired oscillometry and spirometry metrics, indicating that improvements were not incidental but attributable to targeted interventions. Although biologics remain essential for a subgroup of patients with persistent SAD, our data suggest that a substantial proportion can achieve control with optimized inhaled therapy alone. These findings underscore the relevance of *SQA* as a practical and clinically meaningful endpoint and encourage the integration of SAD-specific functional and inflammatory markers in early assessments to personalize treatment and delay or avoid biologic escalation.

### Limitations and Future Perspectives

This study has several limitations. As a single-center, observational investigation with a relatively small sample size (n = 26), the findings may not be broadly generalizable, and the statistical power—particularly in the multivariate analysis—is limited. The lack of a randomized control group and absence of blinding introduces potential selection and interpretation bias. Additionally, although adherence was assessed using validated self-reported questionnaires, objective confirmation via digital monitoring tools was not employed, potentially underestimating the role of adherence variability in clinical outcomes.

Additionally, no formal sample size calculation was performed, as this was an observational, non-interventional study and the primary outcome, persistence of SQA, represents a novel construct without available prior data for effect size estimation. This limits the statistical power of the multivariate analysis and the generalizability of the findings.

Moreover, the limited sample size may have reduced the statistical power of the multivariate analysis, contributing to the lack of significance of otherwise meaningful predictors. Larger sample sizes are likely needed to clarify their independent roles.

Future research should focus on prospective, multicenter studies with larger and more heterogeneous populations to confirm and expand upon these results. Randomized controlled trials comparing single-inhaler triple therapy with biologics in SAD-dominant asthma phenotypes would be particularly valuable. Moreover, longer-term follow-up is needed to determine the stability and durability of the *SQA* state and to assess the potential for completely avoiding the need for biological therapy initiation altogether in selected patients. The use of multivariable models and composite scoring tools may help identify individuals most likely to benefit from inhaled therapy alone. In addition, given that adherence in this study was assessed through self-reported questionnaires, future studies should incorporate objective adherence monitoring tools, such as electronic inhaler tracking or pharmacy refill data, to more accurately quantify adherence and assess its role as a potential confounder. Integrating real-world adherence-tracking technologies, alongside advanced inflammatory and functional biomarkers, could further enhance treatment personalization and identify those most likely to benefit from inhaled therapy alone.

## 5. Conclusions

This real-world study supports the effectiveness of single-inhaler extra-fine triple therapy with beclomethasone–formoterol–glycopyrronium for managing severe asthma with small airway dysfunction. Over one-third of patients achieved sustained disease control without biological therapy, accompanied by significant improvements in symptoms, small airway function, and peripheral inflammation. We introduce Steady Quiet Asthma as a novel, multidimensional treatment goal, clinically comparable to remission, but achievable through inhaled therapy alone. Importantly, baseline indicators such as the %FEV25–75, FEV_1_/FVC ratio, and OCS burden were predictive of this outcome, and the composite model demonstrated excellent accuracy. These findings highlight the value of functional profiling in identifying patients who may achieve long-term control without escalation to biologics, reinforcing the role of optimized inhaled strategies in precision asthma management.

## Figures and Tables

**Table 1:** Baseline characteristics of the enrolled population (n = 26).

Demographic Parameters	
Age (yrs) m ± sd	50.38 ± 14.56
Sex Female n (%)	14 (53.8)
**Lifestyle**	
BMI (kg/m^2^) m ± sd	26.26 ±4.03
Smoke n (%)	
No	19 (59.4)
Ex	13 (40.6)
Pack/year m ± sd	10.17 ± 17.48
**Characteristics of asthma**	
Atopic yes n (%)	26 (80.8)
Duration of disease years m ± sd	21.03 ± 13.33
Age of diagnosis (years) m ± sd	28.65 ± 16.97
Exacerbations last year (n) m ± sd	3.00 ± 3.06
First aid visits last year (n) m ± sd	0.26 ± 0.53
Average reliever usage last month (n) m ± sd	1.19 ± 1.72
OCS cycles last year (n) m ± sd	1.65 ± 1.93
OCS dose last year (prednisone or equivalent mg) m ± sd	18.65 ± 12.92
**Baseline Therapy**	
Course of antibiotics last year n (%)	
0	14 (53.8)
1	9 (34.6)
2	3 (11.5)
LTRA yes n (%)	4 (12.5)
**Comorbidities**	
EGPA yes n (%)	2 (7.7)
Eosinophilic pneumonia yes n (%)	0 (0)
Hypereosinophilic syndrome yes n (%)	0 (0)
Rhinosinusitis yes n (%)	3(11.5)
Nasal polyposis yes n (%)	4 (15.4)
Urticaria yes n (%)	0 (0)
Vocal cord dysfunction yes n (%)	2 (7.7)
COPD yes n (%)	0 (0)
Bronchiectasis yes n (%)	2 (7.7)
GERD yes n (%)	7 (26.9)
OSAS yes n (%)	6 (23.1)
Depressive anxious syndrome yes n (%)	3 (11.5)

Abbreviations: BMI, body mass index; OCS, oral corticosteroids; LTRA, Leukotriene Receptor Antagonist; EGPA, eosinophilic granulomatosis with polyangiitis; GERD, gastro-esophageal reflux; COPD, chronic obstructive pulmonary disease; OSAS, obstructive sleep apnea syndrome; m ± sd, mean ± standard deviation; n, number.

**Table 2:** Comparison of the asthmatic population (n = 26) in open triple-inhaled therapy before and after the introduction of single-device triple-inhaled therapy.

Parameters	T0 Time	T12 Time	*p* Value
ACT m ± sd	16.11 ± 5.60	18.69 ± 3.94	0.059
Average reliver usage last month (n) m ± sd	1.19 ± 1.71	0.50 ± 0.51	0.065
OCS cycle last year MEDIAN IQR	1 (0; 2)	1 (0; 1)	0.005
Mean dosage OCS mg MEDIAN IQR	25 (0; 25)	5 (0; 12.5)	0.001 *
Exacerbations last year n. MEDIAN IQR	2 (1; 4)	2 (0; 2)	0.017 *
Number of visits to emergency room MEDIAN IQR	0 (0; 0)	0 (0; 0)	0.317
Number of course of antibiotic therapy MEDIAN IQR	0 (0; 1)	0 (0; 1)	0.446
%FEV1 m ± sd	68.05 ± 17.63	66.76 ± 20.68	0.740
FEV1 (l) m ± sd	2.05 ± 0.69	2.03 ±0.68	0.877
%FVC m ± sd	80.48 ± 14.17	81.80 ± 14.04	0.624
FVC (l) m ± sd	3.11 ± 0.69	3.07 ± 0.89	0.703
%FEV1/FVC m ± sd	65.55 ± 11.98	65.11 ± 12.41	0.843
%FEV 25–75 m ± sd	48.50 ± 13.86	63.11 ± 12.13	0.000 *
%Rtot m ± sd	149.99 ± 92.81	131.53 ± 40.90	0.331
%TLC m ± sd	106.69 ± 22.19	105.03 ± 21.42	0.650
TLC (l) m ± sd	6.34 ± 1.66	6.22 ± 1.45	0.581
%RV m ± sd	177.94 ± 60.24	145.69 ± 38.82	0.000 *
RV (l) m ± sd	3.49 ± 1.20	2.94 ± 0.00	0.002 *
%RV/TLC m ± sd	155.21 ± 30.34	131.11 ± 32.34	0.000 *
RV/TLC m ± sd	54.10 ± 11.24	51.90 ± 21.28	0.557
R5–20 kPa·L^−1^·s^−1^ MEDIAN IQR	0.14 (0.09; 0.27)	0.09 (0.07; 0.14)	0.031 *
Fres Hz MEDIAN IQR	20.69 (17.61; 24.09)	17.40 (15.00; 19.80)	0.001 *
AX kPa/L MEDIAN IQR	1.25 (0.72; 1.86)	1.44 (0.9; 1.80)	0.354
X5 kPa·L^−1^·s^−1^ MEDIAN IQR	−1.21 (−1.40; −0.9)	−0.80 (−1.02; −0.27)	0.000 *
Eosinophils (n/μL) m ± sd	225.65 ± 204.83	232.38 ± 194.11	0.763
% Eosinophils	3.20 ± 2.53	3.40 ± 2.04	0.553
FeNO 50 (ppb) m ± sd	12.11 ± 13.01	18.61 ± 12.75	0.050 *
FeNO350 (ppb) m ± sd	20.15 ± 13.17	13.15 ± 7.17	0.010 *

Abbreviations: ACT, asthma control test; FEV1, forced expiratory volume in the 1st second; FVC, forced vital capacity; FEF25–75, forced expiratory flow between 25% and 75% of FVC; TLC, total lung capacity; Rtot, total resistance; RV, residual volume; R5–R20, airway resistance from 5 to 20 Hz; Fres, resonance frequency; AX, reactance area; X5, reactance at 5 Hz; Eos, eosinophilia; FeNO 50, fractional exhaled nitric oxide at 50 mL/s; FeNO 350, fractional exhaled nitric oxide at 350 mL/s; m ± sd, mean ± standard deviation; n, number; IQ 25 75, interquartile 25 75; kPa·L^−1^·s^−1^, kiloPascal per liter/second; kPa/L, kiloPascal per liter; Hz, Hertz; kPa·L^−1^·s^−1^, kiloPascal per liter/second ppb, parts per billion. Data are displayed as n (%) or mean ± sd or median IQR. *: significant.

**Table 3:** Baseline comparison between SQA patients and patients who underwent biological therapy after a year.

Parameters	Patients Who Underwent Biological Therapy (n = 17)	SQA Patients (n = 9)	*p* Value
Age (yrs) m ± sd	48.00 ± 19.60	51.11 ± 11.58	0.618
Sex Female n (%)	8 (52.9)	5 (55.6)	0.613
**Lifestyle**			
BMI (kg/m^2^) m ± sd	25.22 ± 4.49	26.82 ± 3.79	0.346
Smoke n (%)			0.402
No	9 (52.9)	6 (6.7)	
Ex	8 (47.1)	3 (33.3)	
Pack/year m ± sd	9.08 ± 14.20	12.22 ± 23.33	0.673
**Characteristics of asthma**			
Atopic yes n (%)	15 (88.2)	6 (66.7)	0.208
Duration of disease years m ± sd	23.64 ± 13.91	16.11 ± 11.24	0.175
Age of diagnosis (years) m ± sd	27.47 ± 14.31	30.88 ± 21.94	0.635
IgE tot	460.20 ± 688.24	624.82 ± 917.93	0.701
**Comorbidities**			
EGPA yes n (%)	2 (11.8)	0 (0)	0.418
Rhinosinusitis yes n (%)	2 (11.8)	1 (11.1)	0.732
Nasal polyposis yes n (%)	3 (17.6)	1 (11.1)	0.569
Vocal cord dysfunction yes n (%)	1 (5.9)	1 (11.1)	0.582
Bronchiectasis yes n (%)	2 (11.8)	0 (0)	0.418
GERD yes n (%)	5 (29.4)	2 (22.2)	0.538
OSAS yes n (%)	2 (11.8)	4 (44.4)	0.084
Depressive anxious syndrome yes n (%)	2 (11.8)	1 (11.1)	0.732

**Table 4:** Clinical, functional, and inflammatory comparison between SQA patients and patients who underwent biological therapy after a year.

	BASELINE			T 12		
	Patients Who Underwent Biological Therapy (n = 17)	SQA Patients (n = 9)	*p* Value	Patients who Underwent Biological Therapy (n = 17)	SQA; Patients (n = 9)	*p* Value
Exacerbations last year (n) m ± sd	3 (1; 4)	1 (0; 2)	0.120	2 (2; 2)	0 (0; 0)	0.000
First aid visits last year (n) m ± sd	0 (0; 1)	0 (0; 0)	0.287	0 (0; 0)	0 (0; 0)	0.000
OCS cycles last year (n) m ± sd	2 (1; 3)	0 (0; 1)	0.002 *	1 (1; 1)	0 (0; 0)	0.000
OCS dose last year (prednisone or equivalent mg) m ± sd	25 (25; 25)	0 (0; 25)	0.031 *	5 (5; 12.5)	0 (0; 0)	0.000
ACT m ± sd	16.35 ± 6.13	15.66 ± 4.76	0.773	16.41 ± 2.69	23 ± 1.5	0.000 *
TAI	52.29 ± 7.03	53.33 ± 1.41	0.668	54 ± 0	53.77 ± 0.66	0.174
%FEV1 m ± sd	60.02 ± 15.50	83.22 ± 9.77	0.000 *	57.70 ± 19.69	83.88 ± 7.57	0.001 *
FEV1 (l) m ± sd	1.87 ± 0.58	2.38 ± 0.81	0.121	1.86 ± 0.62	2.36 ± 0.68	0.088
%FVC m ± sd	75.49 ± 14.33	89.90 ± 7.97	0.003 *	77.88 ± 13.41	89.22 ± 12.70	0.048 *
FVC (l) m ± sd	3.05 ± 0.76	3.23 ± 0.55	0.535	3.06 ± 0.93	3.13 ± 0.87	0.789
%FEV1/FVC m ± sd	61.12 ± 10.26	73.92 ± 10.84	0.010 *	61.00 ± 11.69	72.88 ± 10.19	0.015 *
%FEV 25–75 m ± sd	44.11 ± 14.81	56.77 ± 6.61	0.023 *	58.82 ± 12.33	71.22 ± 6.96	0.010 *
Rtot m ± sd	0.76 ± 0.56	0.99 ± 1.20	0.508	0.46 ± 0.15	0.34 ±0.11	0.039 *
%Rtot m ± sd	163.77 ± 103.86	123.95 ± 64.62	0.308	139.05 ± 41.51	117.33 ± 37.89	0.195
%TLC m ± sd	105.18 ±21.29	109.54 ± 24.87	0.644	103.05 ± 22.70	108.77 ± 19.63	0.512
TLC (l) m ± sd	6.49 ± 1.52	1.96 ± 6.06	0.544	6.32 ± 1.58	6.02 ± 1.24	0.599
%RV m ± sd	180.74 ± 59.13	172.65 ± 65.59	0.761	154.23 ± 40.72	129.55 ± 30.74	0.098
RV (l) m ± sd	3.65 ± 1.11	3.18 ± 1.37	0.369	3.29 ± 0.98	2.27 ± 0.66	0.011 *
%RV/TLC m ± sd	157.79 ± 32.60	150.35 ± 26.66	0.593	144.76 ± 26.82	105.33 ± 26.18	0.002 *
RV/TLC m ± sd	55.25 ± 10.01	51.94 ± 13.65	0.533	50.85 ± 7.21	53.89 ± 36.12	0.237
R5-20 kPa·L^−1^·s^−1^ MEDIAN IQR	0.16 (0.09; 0.27)	0.14 (0.09; 0.15)	0.499	0.12 (0.09; 0.16)	0.05 (0.05; 0.08)	0.009 *
Fres Hz MEDIAN IQR	21 (19.47; 24.58)	17.61 (15.82; 20.63)	0.049 *	18.50 (16.70; 19.96)	14.70 (13.14; 17)	0.014 *
AX kPa/L MEDIAN IQR	1.3 (0.83; 1.86)	0.89 (0.47; 1.3)	0.246	1.64 (1.19; 2.0)	0.90 (0.6; 1.66)	0.043 *
X5 kPa·L^−1^·s^−1^ MEDIAN IQR	−1.20 (−1.4; −0.90)	−1.23 (−1.3; −0.90)	0.807	−0.90 (−1.2; −0.70)	−0.24 (−0.55; −0.14)	0.804
Eosinophils (n/μL) m ± sd	271.88 ± 268.51	176.33 ± 123.46	0.134	271.88 ± 268.51	176.33 ± 123.46	0.227
% Eosinophils	3.91 ± 2.28	2.43 ± 1.00	0.105	3.38 ± 2.82	2.84 ± 1.98	0.078
FeNO 50 (ppb) m ± sd	13.64 ± 15.23	17.66 ± 12.99	0.324	23.76 ± 12.76	8.88 ± 4.48	0.000
FeNO350 (ppb) m ± sd	21.47 ± 13.47	17.66 ± 12.99	0.493	16.94 ± 5.37	6.00 ± 3.87	0.000

Abbreviations: ACT, asthma control test; TAI, test of adherence to inhalers; FEV1, forced expiratory volume in the 1st second; FVC, forced vital capacity; FEF25–75, forced expiratory flow between 25% and 75% of FVC; TLC, total lung capacity; RV, residual volume; Rtot, total resistance; R5–R20, resistance difference between 5 and 20 Hz; Fres, resonance frequency; AX, reactance area; X5, reactance at 5 Hz; FeNO, fractional exhaled nitric oxide; ppb, parts per billion; OCS, oral corticosteroids; m ± sd, mean ± standard deviation; IQ 25–75, interquartile range 25th–75th percentile; SQA, Steady Quiet Asthma. *: significant.

**Table 5:** Univariate and multiple logistic regression analyses of predictors of “Steady Quiet Asthma”

	Univariate Logistic Regression		Multiple Logistic Regression	
	ODD (%CI)	*p* Value	ODD (%CI)	*p* Value
**Demographic Parameters.**				
Age (yrs)	0.982 (0.928; 1.040)	0.538		
Sex female	1.111(0.219; 5.634)	0.899		
**Lifestyle**				
BMI (kg/m^2^)	0.898 (0.722; 1.117)	0.335		
Smoke yes	0.563 (0.105; 3.023)	0.502		
Pack/year	1.010 (0.965; 1.058)	0.660		
**Characteristics of asthma**				
Atopic yes	0.267 (0.035; 2.019)	0.210		
Duration of disease years	0.952 (0.886; 1.022)	0.177		
Age of diagnosis (years)	1.012 (0.965; 1.062)	0.620		
Exacerbations last year (n)	0.857 (0.624; 1.117)	0.340		
First aid visits last year (n)	0.314 (0.037; 2.699)	0.291		
Average reliever usage last month (n)	1.357 (0.842; 2.188)	0.210		
OCS cycles last year (n)	0.124 (0.020; 0.763)	0.024 *	0.186 (0.017; 2.052)	0.170
OCS dose last year (prednisone or equivalent mg)	0.923 (0.856; 0.995)	0.036 *		
**Baseline Therapy**				
Course of antibiotics last year	0.224 (0.043; 1.175)	0.077		
**Comorbidities**				
Rhinosinusitis yes n	0.938 (0.073; 11.99)			
Nasal Polyposis yes n	0.583(0.052; 6.587)	0.571		
**Functional, Oscillometric, and Inflammatory Parameters**				
ACT	0.977 (0.843; 1.133)	0.762		
TAI	1.043 (0.859; 1.267)	0.670		
%FEV1	1.153 (1.029; 1.291)	0.014 *		
FEV1 (l)	3.175 (0.829; 12.166)	0.092		
%FVC	1.143 (1.013; 1.290)	0.030 *		
FVC (l)	1.475 (0.453; 4.804)	0.519		
FEV1/FVC	1.156 (1.012; 1.320)	0.033 *	1.154 (0.962; 1.385)	0.123
%FEV 25–75	1.098 (1.001; 1.201)	0.046 *	1.089 (0.918; 1.292)	0.328
Rtot	1.399 (0.532; 3.679)	0.496		
%Rtot	0.992 (0.977; 1.008)	0.329		
%TLC	0.845 (0.501; 1.427)	0.529		
TLC (l)	1.009 (0.972; 1.047)	0.629		
%RV	0.998 (0.984; 1.012)	0.740		
RV (l)	0.697 (0.327; 1.483)	0.348		
%RV/TLC	0.991 (0.963; 1.020)	0.547		
RV/TLC	0.972 (0.898; 1.051)	0.472		
R5-20 kPa·L^−1^·s^−1^	0.038 (0.000; 41.75)	0.360		
Fres Hz	0.824 (0.664; 1.024)	0.081		
AX kPa/L	0.666 (0.282; 1.575)	0.354		
X5 kPa·L^−1^·s^−1^	0.695 (0.092; 5.253)	0.724		
Eosinophils (n/μL)	0.997 (0.992; 1.003)	0.337		
% Eosinophils	0.912 (0.646; 1.286)	0.598		
FeNO 50 (ppb)	0.965 (0.884; 1.054)	0.429		
FeNO350 (ppb)	0.977 (0.916; 1.042)	0.478		

Abbreviations: BMI = body mass index; OCSs = oral corticosteroids; ACT = asthma control test; TAI = test of adherence to inhalers; FEV_1_ = forced expiratory volume in 1 s; FVC = forced vital capacity; FEV_1_/FVC = FEV_1_-to-FVC Ratio; FEV25–75 = mid-expiratory flow at 25–75%; TLC = total lung capacity; RV = residual volume; Rtot = total resistance; R5–20 = resistance between 5 and 20 Hz; Fres = resonant frequency; AX = area of reactance; X5 = reactance at 5 Hz; FeNO = fractional exhaled nitric oxide. *: significant. Results of univariate and multiple logistic regression analyses exploring the association among clinical, functional, inflammatory, and demographic variables and the achievement of the “Steady Quiet Asthma” outcome. Statistically significant univariate predictors (*p* < 0.05) were assessed for multicollinearity before inclusion in the multivariate logistic regression model.

## Data Availability

The original contributions presented in this study are included in the article. Further inquiries can be directed to the corresponding author(s).
